# Evaluation and Ranking of Researchers – Bh Index

**DOI:** 10.1371/journal.pone.0082050

**Published:** 2013-12-11

**Authors:** D. Gnana Bharathi

**Affiliations:** Knowledge Resource Centre, Central Leather Research Institute, Adyar, Chennai, India; Bar-Ilan University, Israel

## Abstract

Evaluation and ranking of every author is very crucial as it is widely used to evaluate the performance of the researcher. This article proposes a new method, called Bh-Index, to evaluate the researchers based on the publications and citations. The method is built on h-Index and only the h-core articles are taken into consideration. The method assigns value additions to those articles that receive significantly high citations in comparison to the h-Index of the researcher. It provides a wide range of values for a given h-Index and effective evaluation even for a short period. Use of Bh-Index along with the h-Index gives a powerful tool to evaluate the researchers.

## Introduction

There are varied methods used for evaluation of journals and researchers. Journal Impact Factor (JIF) is the most popular and widely used method to evaluate the journals. However, over a period, JIF has been inadvertently used to evaluate the researchers for their performance. This practice is relatively offset by a new method called h-Index that was introduced by Jorge Hirsch [1]. Ranking being in a whole number, ease of calculations and a robust system has enabled the h-Index to become very popular in a short period of time [2].

h-Index is defined by Hirsch as “a scientist has index *h* if *h* of his or her *N*_*p*_ papers have at least *h* citations each and the other (*N*_*p*_ *-h*) papers have *≤ h* citations”. For example, an author who has 5 or more articles with just 5 of them having at least 5 citations, then the author has an h-Index of 5.

However, on critical evaluation of the h-Index it is found that there are a number of issues with the method [3-6]. For example, an author who publishes few articles yet receives thousands of citations is poorly ranked in comparison to those authors of relatively more articles with limited number of citations in each of these individual articles. 

Alternative methods are being proposed by a number of researchers such as g-Index [7], hg-Index [8], A-Index and AR-Index [9,10], R-Index [11] e-Index [12], p-Index [13], P-Index and C-Index [14], etc. These methods were put forth based on identification of specific problems with the h-Index. However, they do not fit for extreme or theoretical situations or they lack simplicity in calculation. For the g-Index, in case of the author with less publications but more citations, citations have to be assigned even to non-existing articles[15]. In the case of A-Index, better scientist is punished for having higher h-Index [16]. Indices such as m-quotient and AR-Index are time dependent.

The proposed new index can be used to evaluate researchers of different age groups or work experience. The index can increase on continuity of citations, even after suspension of publications and provides range of values for a given number of articles and citations.

The basis for the Bh-Index is ranking of the researchers in such a way that the value does not fall below the h-Index nor has a phenomenal increase in case of a few well cited articles. Further, the method is designed to allow researchers with few publications, and more citations, be relatively compared with the authors of large number of publications and limited citations. 

## Method

### Bh-Index

A new methodology to evaluate the researcher, called Bh-Index, is based on threshold value as criteria for the selection of the articles or what is known as ‘h-core articles’. It gives value addition for the articles with very high citations in comparison with threshold value. As a result, author of low h-Index with high level of citations have more impact in comparison to authors of more h-Index with low level of citations. 

In h-Index, each of the qualified articles gets exactly one value, irrespective of the citations. In Bh-Index, these qualified articles gets at least one value. Every article can increase its value by 2, 3, 4…. for doubling, quadrupling, eight times… of the citations over the threshold value – the h-Index. 

The method is similar to the weighted voting system as followed in some voting systems of European Union and United States of America [17]. The difference between the sizes of the countries/states can be just over 10 times larger than the smallest one. But the peak citations of researchers can be more than 100 times of h-Index is common. Therefore, simple proportional values cannot be applied to the evaluation of researchers. The average value or direct proportional value will result in substantial changes in the ranking of the researchers, if such extreme values are equally considered with other articles. Bh-Index addresses these issues.

The excessive citations of the h-core articles have to be rationally valued so that there is no deficit or excessive gain in the ranking of the researchers who have more than h^2^ citations. Considering the extremity in ranking of the researchers, Bh-Index provides a judicial method for ranking the researchers. In the Bh-Index, the value addition is measured on the geometric sequence with ratio of two and the h-Index of the author as the first value.

The method can be expressed as citations of every qualified article factored on the geometric sequence of the h-Index with ratio of two, added together, forms the Bh-Index of the author. 

### Calculation of Bh-Index

As a first step, h-Index is calculated – by ordering citations received by the articles in the descending order. Only those articles in the h-Index, also known as h-core articles, are considered for the calculation of Bh-Index. Making h-Index as the first term, a geometric sequence with ratio of two is derived. The number of citations between the geometric sequences is grouped into mutually exclusive classes. 

A class constitutes *t*_*n*_ to *2t*_*n*_ *-1*. 

Geometric sequence, *t*_*n*_=*t*_1_* *r* ^(n-1)^

Where *n* is the factor, *t*_*1*_ is the first term of factor, *t*_*n*_ is the *n* ^th^ term of factor and *r* is common ratio.

Therefore, *n* ^*th*^ term of the geometric sequence of Bh-Index*=* (*h-Index*)*2 ^*(n-1)*^

Citations of articles falling in the *n* ^*th*^ class of the *n* ^*th*^ term are assigned *n* value. Frequency of the articles in every class is calculated. The factor, *n* is multiplied with the frequency, f of the class. The total is the Bh-Index of the author.

If h-Index is 8, the geometric sequence will be 8, 16, 32, 64…for the classes constituting citations in the range of 8-15, 16-31, 32-63, 64-127….for the factors 1, 2, 3, 4…. Similarly, if h-Index is 15 the geometric sequence will be 15, 30, 60, 120.for the classes constituting citations in the range of 15-29, 30-59, 60-119, 120-239……for the factors 1, 2, 3, 4…. 

h-Index remains as base of the Bh-Index. Researchers with fixed h-Index can increase their Bh-Index, if the articles continue to get more citations to reach each of the benchmarks as 2h, 4h, 8h…. Bh-Index necessitates increase in citations of every article for the increase of each h-Index. For every increase in the h-Index, the benchmarks change to 2(h+1), 4(h+1), 8(h+1)…. 

The citations of every article that reach the benchmarks increase the Bh-Index by one. This enables researchers with given h-Index to show the impact of citations of their articles with reference to that h-Index. If none of the article reaches even the first benchmark, then Bh-Index is equivalent to h-Index. If all the articles meet the first benchmark, the Bh-Index will be 2h, if all the articles meet the second benchmark then Bh-Index will be 3h-Index and so on. 

## Analysis

### Evaluation of researchers

An example to calculate the Bh-Index is given in Table 1. As each of the six articles has six or more citations, h-Index is 6. As a result, the geometric sequence for this case will be 6, 12, 24, 48, 96..… Citation classes will be 6-11, 12-23, 24-47, 48-95…. and factor for the classes will be 1, 2, 3, 4… respectively. The frequency of citations in each of the class is multiplied by the factor of each of the citation class. The values added together give the Bh-Index of 16. 

**Table 1:** Calculation of Bh-Index for the h-Index of 6.

**Articles**	**Citations**	**Geometric sequence**	**Class**	**Factor, *n***	**Frequency, *f***	n*f
1	218	192	192-383	6	1	6
2	34	96	96-191	5	0	0
3	27	48	48-95	4	0	0
4	15	24	24-47	3	2	6
5	8	12	12-23	2	1	2
**6**	**6**	6	6-11	1	2	2
7	4				**Bh-Index**	**16**
8	2					
9	1					
10	0					

**Figure 1:**
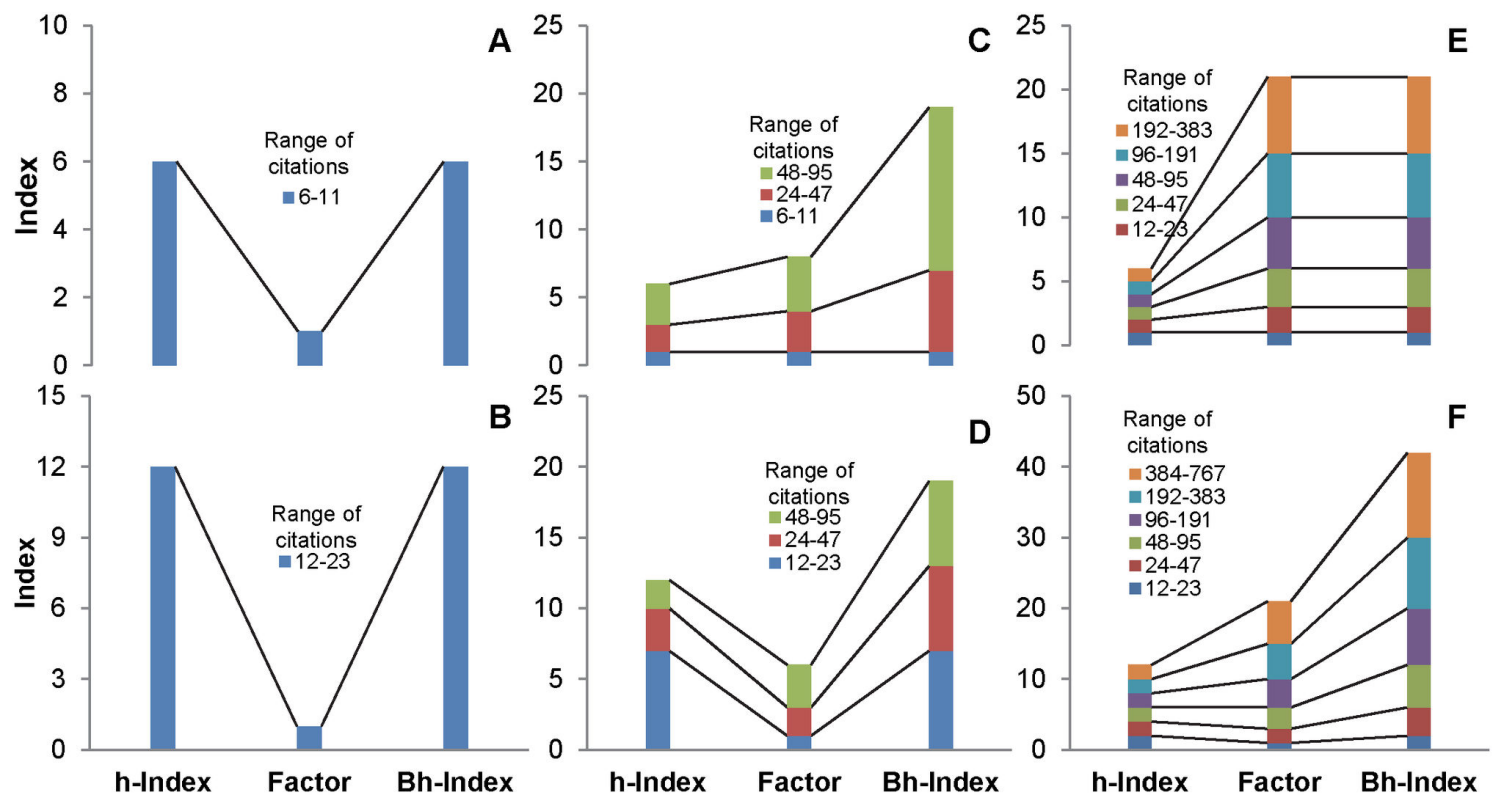
Calculation of Bh-Index from h-Index of 6 and 12.

Figure 1 exhibits how a Bh-Index compares with h-Index. There are three graphs each with h-Index of 6 and 12. As citations in Figure 1A and 1D has one as a factor, Bh-Index is same as h-Index. Figure 1B exhibits that the citations are spread over three different classes of factor 1, 2 and 4. As a result, for the h-Index of 6, Bh-Index is 19. In Figure 1E also citations are in the same factors. However, most of the citations are in the lower class. Hence, h-Index of 12 gets Bh-Index of 19. 

In Figure 1C, citations of the each article follow the geometric sequence. As a result, author with 6 h-Index gets Bh-Index 21. In Figure 1F, though the factors remain same, number of articles and citations has increased. Therefore, the h-Index of 12 gets Bh-Index of 42. It is also clear that the increase in h-Index necessitates more citations for every factor. Calculation of Bh-Index for the Nobel laureates of 2012 is given in Appendix S1.

**Figure 2:**
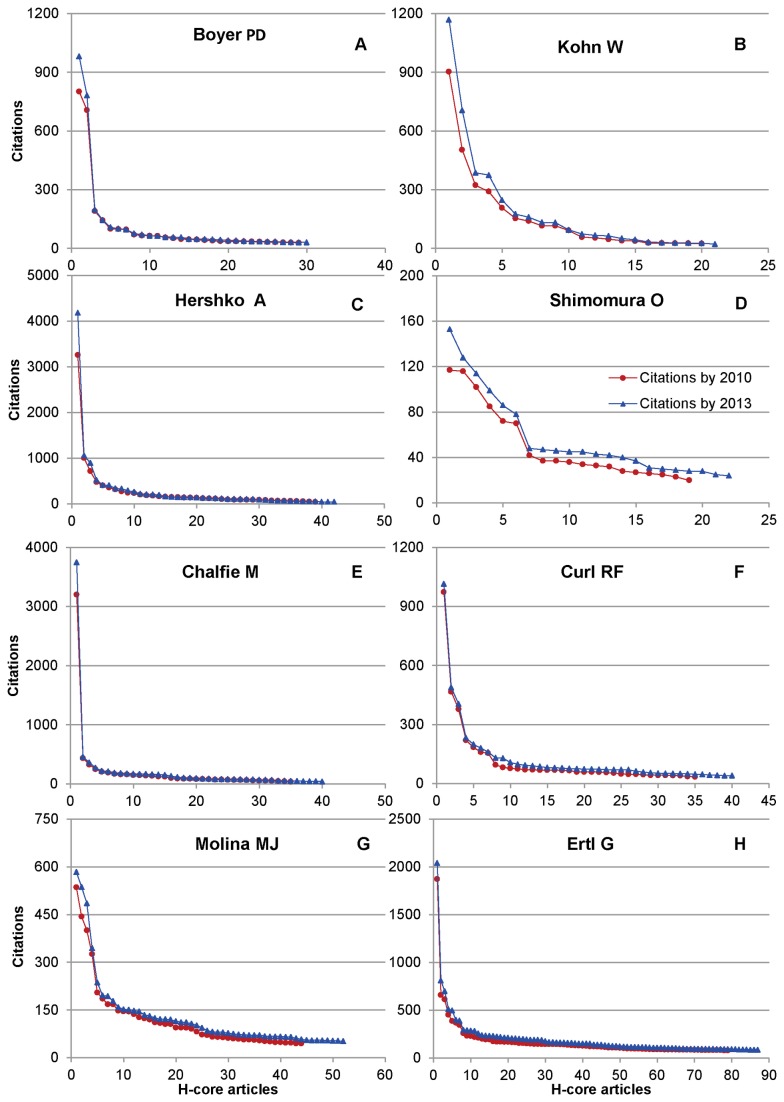
Bh-Index coverage for the h-Index of 2, 10, 25, 50, 75 and 100.

Figure 2 gives Bh-Index for the articles with h-Index of 2, 10, 25, 50 and 100. The lines represent the idealistic conditions from most homogeneous citations to most skewed citations. Each line has sharp bend. The bottom part of the line represents the most skewed citations pattern – one in which only one article get high level of citation and others get less than 2h citations. The top part of the line represents the most homogenous citations where all articles equally reach a given benchmark. The other possibilities of citations fall between the lines.

Bh-Index gives more values for those articles whose citations are equal or cross every benchmark. As increase in the h-Index increases the benchmark, those articles whose citations equaled or crossed the earlier benchmark may need to get more citations to remain in the increased benchmark. Failing to do so will lead to reduction in Bh-Index. This reduction can continue until it equals the h-Index. However, it is unlikely due to the fact that increase in h-Index also necessitates increase in citations of existing articles to certain extent, failing which h-Index remains stagnant.

### Evaluation of researchers over a period of time

**Figure 3:**
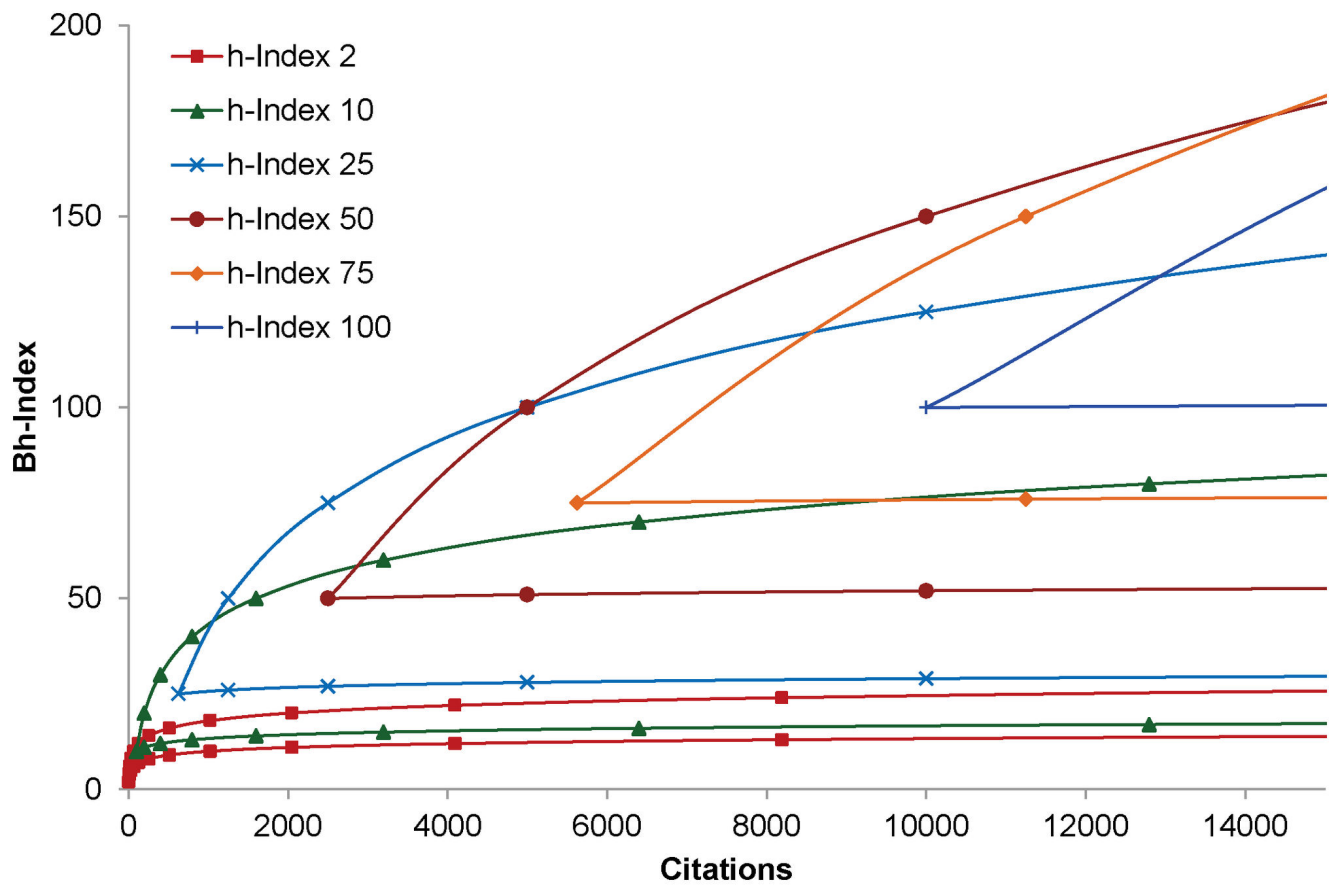
Citations trends of h-core articles in 2010 and 2013.

Citations trends of Nobel laureates in chemistry for 15 years starting from 1995 to 2009 are studied. The citations trends, up to the h-Index of the individual Nobel laureates, are obtained between September 2010 and August 2013 from Web of Science database. The changes in citations trends over the years, for two individuals with identical increase in h-Index are given in Figure 3 and Table 2. 

**Table 2:** Change in h-Index and Bh-Index in 2010 and 2013.

**Name**	**2010**			**2013**			**Difference**		
	**h-core citations**	**h-index**	**Bh-index**	**h-core citations**	**h-Index**	**Bh-Index**	**h-core citations**	**h-Index**	**Bh-Index**
Boyer PD	3103	29	48	3484	30	50	381	1	2
Kohn W	3215	20	53	4050	21	58	835	1	5
Hershko A	10410	39	97	12273	42	99	1863	3	2
Shimomura O	962	19	30	1246	22	37	284	3	7
Chalfie M	7301	35	78	8702	40	85	1401	5	7
Curl RF	4183	35	59	5027	40	68	844	5	9
Molina MJ	5399	44	80	6695	52	83	1296	8	3
Ertl G	13932	79	111	17198	87	128	3266	8	17

Data collected from WoS in September 2010 and August 2013.

In Figure 3A and 3B increase in citations of the researchers resulted in increase of h-Index by one. In Figure 3B, though almost all the articles increased their citations, only five articles have increased their citations to the next level of their respective geometric sequence of the current h-Index. Therefore, Bh-Index increased by five. In Figure 3A, only two articles have increased to the next level of their geometric sequence. Hence, Bh-Index is increased by two.

In Figure 3C, there is an increase of 1863 citations, which is substantially high, in comparison to Figure 3D where citations is increased by 284. Though h-Index increased by three for both the researchers, Bh-Index is increased by two for the former and by seven for the later. This is due to the fact that the geometric sequence of the former starts at 42 while for the later it is 22. As it is clear from the graph, newly added citations in the Figure 3C is concentrated in highly cited articles. There is no clear increase in citations for the poorly cited articles. As the citations are highly skewed, Bh-Index increases by just two values. In Figure 3D every article increased its citations of which citations of seven articles reached the next level. Therefore, Bh-Index increased by seven. 

h-Index, A-Index, e-Index, R-Index, g-Index and gh-Index along with the Bh-Index are given in Table 3. g-Index is not dependent on h-Index and h-core citations and therefore calculated by inclusion of additional citations from the non h-core articles. Other indices are calculated based on h-Index and/or citations. gh-Index is the square root of g and h indices. 

**Table 3:** Indices of Nobel laureates of chemistry from 1995 to 2009.

**Name**	**h-core citations**	**g-Index citations**	**h-Index**	**A-Index**	**e-Index**	**R-Index**	**g-Index**	**gh-Index**	**Bh- Index**
Knowles WS	208	210	4	52.00	13.86	14.42	14	7.48	15
Rose I	518	594	13	39.85	18.68	22.76	24	17.66	22
Tanaka K	645	747	13	49.62	21.82	25.40	27	18.73	28
Skou JC	816	855	10	81.60	26.76	28.57	29	17.03	26
Shimomura O	1246	1428	22	56.64	27.60	35.30	37	28.53	37
Chauvin Y	1951	2273	24	81.29	37.08	44.17	47	33.59	43
Shirakawa H	3093	3798	32	96.66	45.49	55.61	61	44.18	58
Boyer PD	3484	3909	30	116.13	50.83	59.03	62	43.13	50
Rowland FS	3543	4320	40	88.58	44.08	59.52	65	50.99	61
Kohn W	4050	4179	21	192.86	60.07	63.64	64	36.66	58
Yonath AE	4067	4828	34	119.62	53.95	63.77	69	48.44	60
Curl RF	5027	5911	40	125.68	58.54	70.90	76	55.14	68
Molina MJ	6695	8237	52	128.75	63.17	81.82	90	68.41	83
Fenn JB	7783	7853	19	409.63	86.15	88.22	88	40.89	62
Chalfie M	8702	9124	40	217.55	84.27	93.28	95	61.64	85
Ramakrishnan V	10236	11421	53	193.13	86.18	101.17	106	74.95	101
Hershko A	12273	12634	42	292.21	102.51	110.78	112	68.59	99
Kroto HW	13003	15673	66	197.02	92.99	114.03	125	90.83	110
Zewail A	13039	16164	81	160.98	80.49	114.19	127	101.42	113
Walker JE	13398	16019	66	203.00	95.09	115.75	126	91.19	110
Schrock RR	13446	16605	81	166.00	82.98	115.96	126	101.02	110
Kornberg RD	15927	19076	80	199.09	97.61	126.20	138	105.07	125
Macdiarmid AG	16976	20330	76	223.37	105.83	130.29	142	103.88	136
Ertl G	17198	21545	87	197.68	98.13	131.14	146	112.70	128
Crutzen PJ	17366	20277	78	222.64	106.22	131.78	142	105.24	127
Agre P	17441	20875	81	215.32	104.31	132.06	144	108.00	131
Ciechanover A	18533	20929	59	314.12	122.69	136.14	144	92.17	123
Noyori R	23768	27063	84	282.95	129.27	154.17	164	117.37	161
Steitz TA	24465	27773	83	294.76	132.57	156.41	166	117.38	159
Mackinnon R	25132	26524	70	359.03	142.24	158.53	162	106.49	161
Wuthrich K	30479	36892	98	311.01	144.48	174.58	192	137.17	161
Sharpless KB	31864	36171	83	383.90	158.03	178.50	190	125.58	159
Pople JA	33237	35129	63	527.57	171.08	182.31	187	108.54	160
Grubbs RH	34663	41724	101	343.20	156.40	186.18	204	143.54	180
Tsien RY	43721	49052	108	404.82	179.04	209.10	221	154.49	216
Heeger AJ	50485	61147	118	427.84	191.21	224.69	247	170.72	213
Smalley RE	54978	60824	119	462.00	202.03	234.47	246	171.10	233

Data collected in August 2013.

A-Index gives high values whenever the h-Index is small. As e-Index is square root of excess citations of the h-core articles, its values can widely vary from zero to infinity. Researchers with more h-core citations have high R-Index. g-Index also follows the same method, based on inclusion of increased number of citations. The Bh-Index on the other hand, measures by evaluating the individual h-core articles, based on its citations. Thus, it gives more appropriate values.

## Discussion

**Figure 4:**
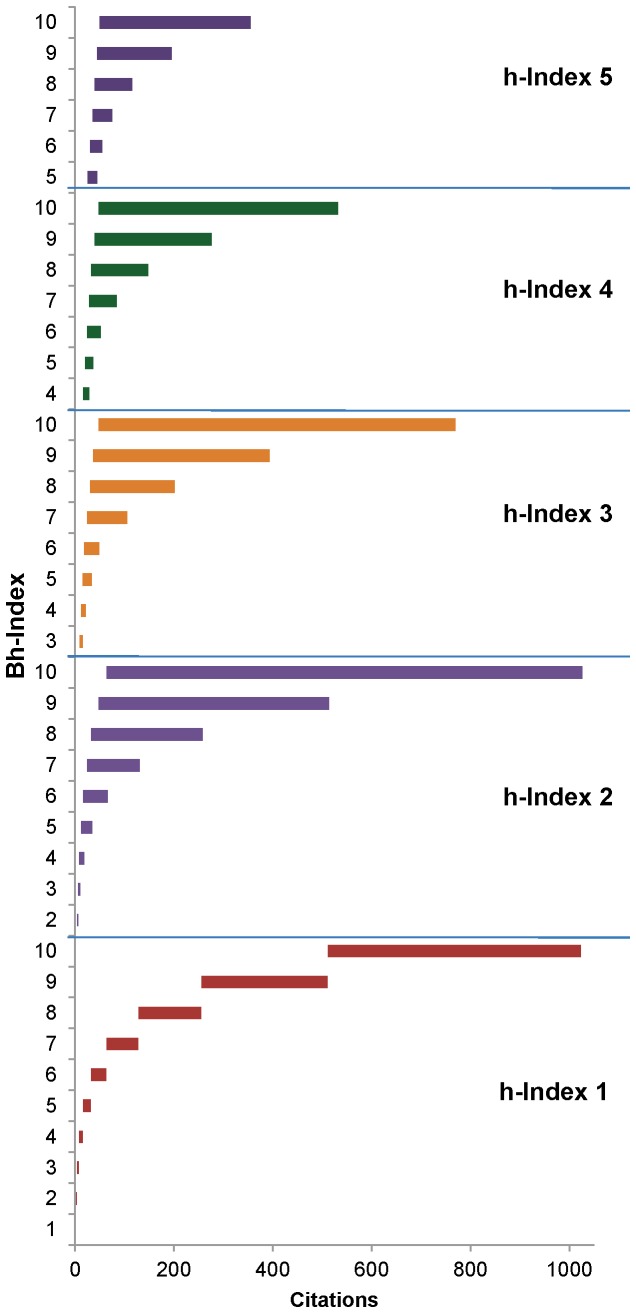
Bh-Index for extreme level of citations for h-Index of 1, 2, 3, 4 and 5.

Bh-Index has a range of values for the publication of articles and their citations. Range of Bh-Index for given h-Indexes for various citation ranges is given in the Figure 4. For easy understanding h-Index of 1, 2, 3, 4, and 5 are considered. Author with only one article can have more Bh-index, if the article continues to receive citations. As Bh-Index follows geometric sequence, double citations are necessary for every additional increase in Bh-Index. Author with h-Index of 2 can get Bh-Index of 16, if each of the articles gets citations in the range of 256 to 511. However, if one article is cited between 2 to 3 and other is cited in between 256 to 511, Bh-Index will be 9. 

If one author with large number of citations from a few articles has Bh-Index for a given h-Index, other author can make the same Bh-Index with relatively lower level of citations, if all articles are equally cited. For example, author of h-Index of 4 can get Bh-Index of 8, if only one article gets citations between 64 and 127 and others get citations in between 4 to 7. Other researchers can have same Bh-Index for the same h-Index if all four articles get citations in between 8 and 15. If the first author continues to receive citations only from one article, the Bh-Index will be 12, if the citations of the article are between 1024 and 2048. On the other hand, the second author can get Bh-Index of 12 if all four articles get citations between 16 and 31. 

Therefore, the author with high h-Index can score more Bh-Index when more articles get high citations. If citations are confined to fewer of articles, even if the total number of citations is equal, the Bh-Index will be low. Author with low h-Index has to have more citations from most of the articles to have high Bh-Index. If no article gets double or more citations than the h-Index, Bh-Index equals the h-Index. This characteristic enables young researcher with few articles having high citations to be compared with the experienced author with more articles. 

For every increase in h-Index, the author needs to get additional citations from the non h-core articles. The authors may also have to get increase in citations from the lowest cited article(s) that are already included in the h-Index. In case of Bh-Index, for every increase in the h-Index, there has to be increase in citations of almost all the articles. This is essential to retain the Bh-Index, if the h-Index increases, even by one value.

As a result, citations of an article that received *n* factor become *n-1* factor, if the number of citations of the article falls below the factor. Therefore, if the author who fails to increase the citations of all the articles, especially those close to the lower limit of the class, Bh-Index will decrease, marginally, for every increase in h-Index. This may prevent self-citations to boost the h-Index, to certain extent, if both the indices are used to evaluate the researchers. 

For example, a researcher has h-Index of 3 with citations as 4, 6 and 12. As per the calculations of geometric sequence 3, 6, 12.…Bh-Index will be 6. Let us assume the researcher's one more article received 4 citations. Hence, the citations are 4, 4, 6 and 12. Accordingly, the h-Index is 4. However, as per the redefined geometric sequence of 4, 8, 16…. Bh-Index will be 5. 

## Conclusion

Bh-Index provides a comprehensive measurement to evaluate the researcher. It gives a range of values for the citations for the h-core articles. While h-Index evaluates the overall performance by fixing the benchmark for the author, Bh-Index evaluates the individual articles of the h-Index, as well as the author, effectively. 

For every increase of h-Index, citations of marginal articles of the existing h-core articles may be necessary. In such cases, if the marginal articles fail to increase their citations, h-Index remains stagnant. For every increase of h-Index, Bh-Index demands increase in citations of all the articles. For every article that fails to reach the new benchmark from the existing one, Bh-Index is reduced by one. On the other hand, any article that reaches every additional benchmark increases the Bh-Index by one, irrespective of the increase in h-Index. 

More citations from every h-core article increases the Bh-Index for reaching every benchmark of the geometric sequence. Bh-Index of author of more publications and h-Index with skewed citations can be equaled by an author with less publications and h-Index, if most of the articles receive high citations. 

Further, Bh-Index takes into consideration the citations of every qualified article and ranks accordingly. Similar to h-Index, use of the Bh-Index alone will not show the true picture of the citation behavior of the articles of the researcher. However, if associated with the h-Index it will prove the true characteristics of citations and real impact of the researchers.

## Supporting Information

Appendix S1 **Examples of calculation of Bh-Index for seven Nobel Laureates of 2012.** The data were collected in the month of April 2013 from Web of Science database.
(DOCX)
