# Supplementary material for: Evaluation and Ranking of Researchers – Bh Index
Source: PLoS One. 2013 Dec 11;8(12):e82050. doi: 10.1371/journal.pone.0082050 (PMC3859578; doi:10.1371/journal.pone.0082050)
Supplement: Appendix S1 — Examples of calculation of Bh-Index for seven Nobel Laureates of 2012. The data were collected in the month of April 2013 from Web of Science database. (DOCX) [file pone.0082050.s001.docx]

**Examples of calculation of Bh-Index for Nobel Laureates of 2012**

(data collected in April 2013 from WoS)

**1. Lefkowitz RJ**

h-index 155

Citations from h-core articles:

1259, 1154, 980, 970, 829, 810, 749, 745, 744, 714, 702, 690, 671, 665, 626, 604, 580, 567, 564, 559, 546, 543, 540, 535, 507, 494, 489, 465, 463, 462, 461, 459, 445, 440, 436, 436, 434, 428, 418, 414, 411, 410, 401, 391, 389, 387, 385, 382, 377, 371, 370, 369, 368, 365, 363, 360, 359, 357, 356, 355, 354, 346, 344, 343, 338, 337, 325, 317, 314, 313, 310, 309, 305, 304, 303, 302, 299, 294, 290, 287, 286, 286, 283, 278, 278, 271, 271, 270, 264, 260, 259, 257, 255, 243, 241, 240, 240, 238, 234, 230, 229, 229, 228, 222, 221, 220, 219, 218, 216, 214, 214, 214, 208, 207, 206, 205, 203, 203, 200, 199, 197, 197, 196, 196, 193, 193, 192, 191, 190, 189, 188, 187, 185, 184, 183, 181, 181, 181, 180, 175, 173, 172, 170, 170, 169, 166, 165, 164, 162, 161, 161, 161, 161, 158, 158.

Calculation of Bh-Index:

| **Geometric sequence** | **Groups** | **Factor, n** | **Frequency, f** | **n*f** |
| --- | --- | --- | --- | --- |
| 2480 |  |  |  |  |
| 1240 | 1240-2439 | 4 | 1 | 4 |
| 620 | 620-1239 | 3 | 14 | 42 |
| 310 | 310-619 | 2 | 56 | 112 |
| 155 | 155-309 | 1 | 84 | 84 |
|  |  |  | **Bh-Index** | **242** |

**2. Kobilka BK**

h-Index 81

Citations from h-core articles:

1387, 877, 745, 690, 619, 604, 580, 567, 546, 543, 463, 462, 416, 386, 385, 385, 312, 311, 299, 287, 285, 269, 264, 256, 255, 255, 253, 246, 243, 240, 228, 225, 223, 223, 216, 211, 209, 207, 206, 195, 192, 182, 178, 171, 162, 162, 161, 160, 160, 157, 157, 157, 156, 151, 149, 145, 135, 130, 129, 127, 126, 120, 117, 117, 116, 113, 109, 109, 107, 100, 97, 95, 95, 95, 92, 91, 89, 88, 88, 88, 82.

Calculation of Bh-Index:

| **Geometric sequence** | **Groups** | **Factor, n** | **Frequency, f** | **n*f** |
| --- | --- | --- | --- | --- |
| 2592 |  |  |  |  |
| 1296 | 1296-291 | 5 | 1 | 5 |
| 648 | 648-1295 | 4 | 3 | 12 |
| 324 | 324-647 | 3 | 12 | 36 |
| 162 | 162-323 | 2 | 30 | 60 |
| 81 | 81-161 | 1 | 35 | 35 |
|  |  |  | **Bh-Index** | **148** |

**3. Haroche S**

h-Index 48

Citations from h-core articles:

1142, 1032, 574,562, 523, 493, 429, 427, 368, 344, 343, 251, 250, 242, 240, 227, 222, 206, 195, 182, 175, 170, 156, 156, 147, 145, 140, 137, 137, 118, 115, 105, 103, 101, 85, 84, 84, 83, 80, 72, 71, 70, 67, 67, 66, 59, 58, 55.

Calculation of Bh-Index:

| **Geometric sequence** | **Groups** | **Factor, n** | **Frequency, f** | **n*f** |
| --- | --- | --- | --- | --- |
| 1536 |  |  |  |  |
| 768 | 768-1535 | 5 | 2 | 10 |
| 384 | 384-767 | 4 | 6 | 24 |
| 192 | 192-383 | 3 | 11 | 33 |
| 96 | 96-191 | 2 | 15 | 30 |
| 48 | 48-95 | 1 | 14 | 14 |
|  |  |  | **Bh-Index** | **111** |

**4. Wineland DJ**

h-Index 64

Citations from h-core articles:

983, 834, 785, 729, 640, 582, 562, 473, 464, 456, 423, 421, 421, 421, 416, 399, 381, 364, 356, 349, 309, 306, 274, 254, 246, 224, 219, 211, 208, 198, 191, 186, 178, 171, 151, 151, 147, 146, 145, 141, 124, 122, 122, 122, 120, 115, 107, 103, 101, 101, 99, 94, 89, 88, 87, 74, 74, 72, 72, 70, 70, 68, 66, 66.

Calculation of Bh-Index:

| **Geometric sequence** | **Groups** | **Factor, n** | **Frequency, f** | **n*f** |
| --- | --- | --- | --- | --- |
| 1024 |  |  |  |  |
| 512 | 528-1055 | 4 | 7 | 28 |
| 256 | 264-527 | 3 | 16 | 48 |
| 128 | 132-263 | 2 | 17 | 34 |
| 64 | 66-131 | 1 | 24 | 24 |
|  |  |  | **Bh-Index** | **134** |

**5. Gurdon JB**

h-Index 48

Citations from h-core articles:

396, 324, 321, 305, 261, 259, 244, 234, 224, 179, 171, 155, 153, 145, 143, 141, 129, 123, 121, 120, 119, 119, 113, 112, 107, 106, 104, 97, 81, 81, 81, 79, 76, 76, 75, 75, 68, 68, 66, 64, 57, 55, 55, 53, 51, 49, 48, 48.

Calculation of Bh-Index

| **Geometric sequence** | **Groups** | **Factor, n** | **Frequency, f** | **n*f** |
| --- | --- | --- | --- | --- |
| 768 |  |  |  |  |
| 384 | 384-767 | 4 | 1 | 4 |
| 192 | 192-383 | 3 | 8 | 24 |
| 96 | 96-191 | 2 | 19 | 38 |
| 48 | 48-95 | 1 | 20 | 20 |
|  |  |  | Bh-Index | 86 |

**6. Yamanaka S**

h-Index 63

Citations from h-core articles:

4839, 3906, 1597, 1349, 970, 759, 713, 456, 429, 397, 285, 272, 260, 255, 245, 239, 239, 238, 222, 209, 208, 205, 198, 196, 196, 174, 173, 172, 171, 169, 161, 158, 153, 139, 134, 132, 130, 120, 117, 105, 105, 98, 96, 94, 93, 85, 84, 82, 81, 81, 72, 69, 69, 69, 68, 68, 68, 67, 66, 65, 64, 63, 63.

Calculation of Bh-Index

| **Geometric sequence** | **Groups** | **Factor, n** | **Frequency, f** | **n*f** |
| --- | --- | --- | --- | --- |
| 8064 |  |  |  |  |
| 4032 | 4032-8063 | 7 | 1 | 7 |
| 2016 | 2016-4031 | 6 | 1 | 6 |
| 1008 | 1008-2015 | 5 | 2 | 10 |
| 504 | 504-1007 | 4 | 3 | 12 |
| 252 | 252-503 | 3 | 7 | 21 |
| 126 | 126-251 | 2 | 23 | 46 |
| 63 | 63-125 | 1 | 26 | 26 |
|  |  |  | **Bh-Index** | **128** |

**7. Whitesides G**

h-Index 168

Citations from h-core articles:

2976, 2909, 2392, 2275, 2195, 2132, 2007, 1613, 1413, 1395, 1237, 1187, 1177, 1107, 1098, 1017, 966, 952, 932, 880, 877, 867, 833, 824, 78, 771, 711, 709, 706, 694, 691, 686, 674, 649, 606, 581, 569, 554, 549, 542, 530, 522, 519, 510, 465, 445, 439, 422, 410, 410, 405, 401, 399, 399, 388, 382, 382, 373, 371, 364, 361, 359, 359, 358, 346, 341, 341, 340, 331, 330, 327, 322, 319, 315, 313, 309, 306, 301, 301, 299, 299, 295, 293, 289, 289, 286, 284, 282, 282, 278, 277, 276, 275, 269, 262, 255, 254, 253, 252, 249, 246, 246, 246, 241, 238, 238, 235, 235, 235, 235, 234, 234, 233, 229, 228, 227, 222, 217, 215, 214, 212, 211, 210, 210, 208, 207, 204, 203, 203, 202, 202, 201, 201, 201, 201, 200, 198, 197, 196, 196, 195, 195, 194, 194, 193, 191, 188, 186, 184, 184, 183, 183, 182, 182, 178, 177, 174, 174, 174, 173, 173, 172, 172, 170, 169, 168, 168.

Calculation of Bh-Index

| **Geometric sequence** | **Groups** | **Factor, n** | **Frequency, f** | **n*f** |
| --- | --- | --- | --- | --- |
| 5344 |  |  |  |  |
| 2672 | 2688-5376 | 5 | 2 | 10 |
| 1336 | 1344-2687 | 4 | 8 | 32 |
| 668 | 672-1343 | 3 | 23 | 69 |
| 334 | 336-671 | 2 | 35 | 70 |
| 167 | 168-335 | 1 | 99 | 99 |
|  |  |  | **Bh-Index** | **280** |
